# Chronic Isolated Fallopian Tube Torsion in a Sexually Inactive Adolescent Female Diagnosed Peroperatively

**DOI:** 10.1155/2024/2581337

**Published:** 2024-01-22

**Authors:** Yuhya Hirahara, Koichi Nagai, Kazunori Mukaida

**Affiliations:** ^1^Department of Obstetrics and Gynecology, Yokohama City University Graduate School of Medicine, Yokohama, Japan; ^2^Department of Obstetrics and Gynecology, National Hospital Organization Yokohama Medical Center, Yokohama, Japan

## Abstract

**Introduction:**

Isolated fallopian tube torsion (IFTT) has rarely been confirmed in sexually inactive adolescents, and preoperative diagnosis of IFTT is difficult because of the absence of specific symptoms. Therefore, pediatric patients with IFTT tend to be misdiagnosed before the surgery.

**Case:**

A 15-year-old female patient with no history of abdominal surgery or sexual intercourse presented with acute left lower abdominal pain and purpura. MRI revealed hydrosalpinx in the left adnexal region. Her abdominal pain had completely resolved at our examination; she was followed up as an outpatient. One month after the initial presentation, she experienced a large volume of watery discharge. Magnetic resonance imaging, which was performed every three months, showed a gradual decrease in the size of the hydrosalpinx; however, it persisted in the left adnexal region. She was counseled to receive laparoscopy to treat the hydrosalpinx, which was the most likely cause of the watery discharge. IFTT was detected during the laparoscopy, and left salpingectomy was performed for pathological evaluation of the persistent hydrosalpinx. Following laparoscopy, the patient's watery discharge was resolved. Pathological findings confirmed no signs of malignancy.

**Conclusion:**

Our current report highlighted watery discharge as an indicative symptom of IFTT. It is unclear whether IFTT induced the hydrosalpinx or vice versa. We presumed that the patient's hydrosalpinx occurred due to IFTT, because the patient complained watery discharge one month after the initial appearance, and noncongenital hydrosalpinx in adolescents, especially without a history of sexual intercourse, is a rare event. Clinicians should consider IFTT in patients presenting with unremitting watery discharge and hydrosalpinx, because IFTT may persist even after the pain disappears.

## 1. Introduction

Isolated fallopian tube torsion (IFTT) is defined as a rotation of the fallopian tube around its longitudinal axis without ovarian involvement. This may have occurred in approximately one in 1,500,000 women [1]. Almost 80% of IFTT cases are in fertile women, and it is a rare cause of acute abdomen premenarche [2]. In this group, congenital malformations are a possible risk factor for IFTT [3]. IFTT has various nonspecific symptoms; however, abnormal vaginal discharge has not been previously reported [1]. IFTT has no clinical manifestations or laboratory findings [1]. Imaging tests, including ultrasonography (US) and magnetic resonance imaging (MRI), may not be sufficient to make a definite diagnosis [4]. Surgery is the definitive therapy, and early diagnosis and treatment are essential for salvaging fallopian tube function in women of reproductive age. Herein, we report the case of a patient with IFTT who presented with watery discharge after complete resolution of acute abdominal pain and who was diagnosed laparoscopically.

## 2. Case Report

A 15-year-old woman with purpura presented to her primary care physician who prescribed betamethasone dipropionate ointment for a provisional diagnosis of immunoglobulin A (IgA) vasculitis. One week after the initial examination, she visited the pediatric outpatient clinic at our hospital complaining of sudden pain in the left lower abdominal area. At presentation, she experienced no fever, nausea, vomiting, or diarrhea and had no history of metrorrhagia or abnormal vaginal discharge. There was no history of abdominal surgery or sexual intercourse. Her menstrual periods were usually regular and without complications, including dysmenorrhea or menorrhagia. She had been receiving medications for schizophrenia—olanzapine, suvorexant, sodium valproate, and paliperidone were administered daily; risperidone and biperiden were administered as needed.

**Figure 1:**
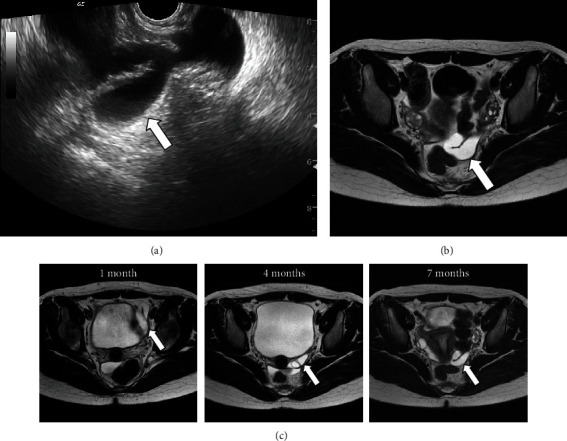
(a) Transrectal ultrasound appearance of a cystic mass (arrow). (b) Axial T2-weighted magnetic resonance imaging (MRI) at the first hospitalization revealing a normal ovary and a hydrosalpinx (arrow). (c) The hydrosalpinx shows a gradual reduction in size (arrows) on MRI.

On examination, her abdomen was soft, but tender in the left lower quadrant. No signs of peritoneal irritation were noted. She had a brown rash on the chest and abdomen. After admission, transabdominal US revealed left hydronephrosis and a dilated tubal structure on the left side of the bladder, while contrast-enhanced computed tomography (CT) showed a suspected left adnexal tumor. In the evaluation at our obstetrics and gynecology department, her abdominal pain had completely resolved, and the transrectal US showed a cystic structure and normal uterus and ovaries (Figure 1(a)). MRI revealed a tortuous tubular structure in the left pelvis, with the inner part of the lesion showing a water-like density, with no signs of malignancy (Figure 1(b)). Laboratory test results were unremarkable except for a slightly elevated white blood cell count (9100/*μ*L). Therefore, the patient was diagnosed with hydrosalpinx, which her abdominal pain was attributed to. The pain completely resolved thereafter, and we decided to treat her conservatively as an outpatient.

Her abdominal pain continued to diminish; however, she complained of a large volume of watery discharge one month after the initial presentation. MRI, which was performed every three months during the observation period, showed a gradual decrease in the size of the hydrosalpinx (Figure 1(c)); hence, we continued to monitor her symptoms carefully. Nine months after her first admission, the left hydrosalpinx had not completely disappeared, and watery discharge continued. Therefore, we counseled the patient about laparoscopy as a treatment for the hydrosalpinx, which was the most likely cause of the watery discharge.

**Figure 2:**
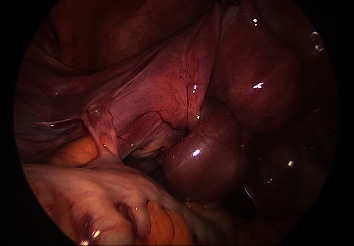
Torsion of the left fallopian tube (laparoscopic intraoperative image).

**Figure 3:**
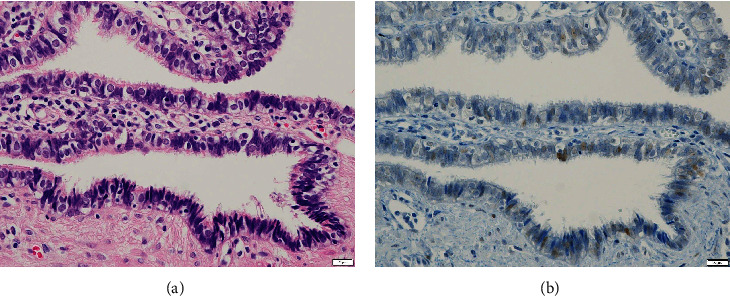
(a) High-power field of hematoxylin and eosin-stained resected left fallopian tube. (b) Immunohistochemistry for p53 showing focal and very weak staining. There is no evidence of malignancy.

We performed laparoscopic surgery using three ports (one umbilical and two lateral ports in the lower abdomen). Intra-abdominal observation revealed a left fallopian tube with torsion (360° clockwise rotation) and swelling of the entire tube (Figure 2). Although no sign of malignancy was indicated by the series of MRI, pathological evaluation was required to determine the cause of the persistent tubal cystic tumor. Moreover, there was a high risk of recurrent torsion due to continuous hydrosalpinx. Based on the patient's condition, we decided to perform a left salpingectomy. The total surgical duration was 40 minutes, and blood loss was minimal. The patient's postoperative course was uneventful. She had no further watery discharge. Histopathological examination confirmed the hydrosalpinx. Focal nuclear hyperchromasia was observed; however, the tubal epithelial structures were preserved (Figure 3(a)). Immunohistochemistry showed nonspecific weak staining for p53, with no evidence of malignancy (Figure 3(b)).

## 3. Discussion

The proposed etiology of IFTT can be divided into intrinsic and extrinsic factors [4]. Intrinsic factors include congenital abnormalities (e.g., long fallopian tube), hydrosalpinx, hematosalpinx, tubal neoplasm, and a history of tubal ligation. Extrinsic factors include ovarian or paraovarian masses, pelvic adhesions, pelvic venous congestion, sudden body movement, and uterine enlargement. IFTT in adolescents is mainly associated with intrinsic causes [3, 5], and tubal torsion occurs more frequently on the right side [5]. This tendency is reportedly caused by reduced mobility of the left tube owing to the sigmoid colon. Another reason is that patients with right lower abdominal pain often undergo surgery for suspected appendicitis.

IFTT has no specific symptoms. The most common manifestation is nonspecific abdominal pain, indicating that IFTT cannot be distinguished from other diseases that cause acute abdominal pain. One Italian multicenter retrospective study analyzed the characteristics of 20 surgically diagnosed IFTT cases [6]. They reported that 13 patients (65%) presented with a history of abdominal pain for >24 hours after symptom onset; the median time from onset to evaluation was 3.4 days in 11 patients, and two patients had recurrent pain. They also reported that 12 patients (60%) had nausea, and ten patients (50%) had vomiting. Another IFTT case series reported similar results [1]; ten patients, under 18 years of age, were reported to have confirmed IFTT. All patients had abdominal pain; the mean duration from onset to admission was 4.97 days, five (50%) had nausea, and five (50%) had vomiting. None of the patients had an abnormal discharge.

IFTT has no pathognomonic imaging signs. US is the most commonly used detection modality. The representative findings are dilated fallopian tubes with thickened walls and tubal beak signs, which are dilated tubular structures with tapered ends [7]. These can all point to a diagnosis of IFTT. Evaluation of blood flow using color Doppler imaging is useful. Physicians can suspect IFTT with the presence of the whirlpool sign over the twisted vasculature; however, this is not a common presentation [4]. CT can detect other causes of acute abdomen, such as appendicitis [7]. MRI may determine the probability of IFTT, particularly in pregnant women [8]. However, these modalities are not sufficient for definitive diagnosis, and direct observation with laparoscopy is the gold standard for diagnosis.

In some cases, hydrosalpinx pathology is associated with IFTT. Bertozzi et al. reported that six of 20 patients had IFTT associated with hydrosalpinx [6]. Coutureau et al. [9] also identified that 37% (6/16) of patients with IFTT displayed hydrosalpinx through CT or MRI examinations. However, hydrosalpinx is rare in adolescents, especially in those without a history of pelvic inflammation [10]. It is unclear whether hydrosalpinx is a causative agent or a consequence of IFTT.

Pediatric patients with IFTT tend to be misdiagnosed preoperatively more often than adult patients. Raban et al. reported that the preoperative IFTT detection rates in adolescents and adults were 18.2% and 37.5%, respectively [4]. They considered that this tendency was due to the population's low propensity for IFTT and limited imaging methods. Most cases of IFTT are reported among women during their fertile period, and a lower fertile patient population in adolescents leads to low familiarity and low suspicion. Additionally, transabdominal US is preferable for pediatric patients, although this might reduce the ability to visualize the deep pelvic structures. Thus, the accurate preoperative diagnosis rate of IFTT in adolescents is low.

Surgery is the standard therapy, and several surgical options have been reported. Salpingectomy, salpingotomy, and detorsion are all therapeutic options for IFTT [1]. Tubal clip occlusion has been reported as an alternative to salpingectomy [11]. These therapeutic methods can be selected based on the patient's age, the status of the included fallopian tube, and the purpose of treatment (e.g., tubal malignancy is suspected). In the context of fertility, it is also important to consider patient's factors, including expected treatment costs for infertility, patients' safety, and individual preferences, to decide whether to perform tubal reconstruction or salpingectomy [12]. Also, Obrzut et al. highlighted the advantages and disadvantages of tubal reconstruction and salpingectomy for distal tubal disease [12]. Clinicians are required to make balancing of those factors in making decisions on the treatment method for IFTT through adequate communication with patients. Additionally, laparoscopy is the gold standard for establishing a diagnosis of IFTT. Therefore, early laparoscopic intervention should be considered to offer enough therapeutic options when a patient, suspected of having IFTT, undergoes a pathological change that is associated with IFTT, such as hydrosalpinx.

## 4. Conclusion

In this present report, we reported the case of a 15-year-old girl with no history of sexual intercourse who had developed IFTT associated with hydrosalpinx. Whether the hydrosalpinx was a causative factor of IFTT or its consequence is unclear. However, we presumed that the patient's hydrosalpinx occurred due to IFTT, because she had never experienced watery discharge before the current episode, and hydrosalpinx in adolescents, without a history of sexual intercourse, is a rare event.

To the best of our knowledge, this is the first report that highlights watery discharge, although it is not a frequent symptom of IFTT, as an indicative symptom of suspected IFTT in patients with a history of acute abdominal pain. Physicians should carefully consider the probability of IFTT when patients have unremitting abnormal discharge and hydrosalpinx even after the pain disappears. Laparoscopic surgery is helpful in both diagnosis and treatment. Furthermore, laparoscopy is especially suitable for adolescents because it is comparatively less invasive than laparotomy.

## Data Availability

Data sharing is not applicable to this article as no datasets were generated or analyzed during the current study.

## Supplementary Materials

Supplementary materials The CARE checklist for case reports.
