# Supplementary material for: Chronic Isolated Fallopian Tube Torsion in a Sexually Inactive Adolescent Female Diagnosed Peroperatively
Source: Case Rep Surg. 2024 Jan 22;2024:2581337. doi: 10.1155/2024/2581337 (PMC10824574; doi:10.1155/2024/2581337)
Supplement: Supplementary materials — The CARE checklist for case reports. [file 2581337.f1.pdf]

CARE
case report guidelines

CARE Checklist of information to include when writing a case report

(cc) BY-NC-ND

Topic	Item	Checklist item description	Reported on Line
**Title**	1	The diagnosis or intervention of primary focus followed by the words "case report"	Page #1, lines #190–202
**Key Words**	2	2 to 5 key words that identify diagnoses or interventions in this case report, including "case report"	Page #3, lines #69
**Abstract (no references)**	3a	Introduction: What is unique about this case and what does it add to the scientific literature?	Page #3, lines #46–49
	3b	Main symptoms and/or important clinical findings	Page #3, lines #50–56
	3c	The main diagnoses, therapeutic interventions, and outcomes	Page #3, lines #56–60
	3d	Conclusion—What is the main "take-away" lesson(s) from this case?	Page #3, lines #61–67
**Introduction**	4	One or two paragraphs summarizing why this case is unique (**may include references**)	Page #5, lines #71–83
**Patient Information**	5a	De-identified patient specific information.	Page #5, lines #86–95
	5b	Primary concerns and symptoms of the patient.	Page #5, lines #86–95
	5c	Medical, family, and psycho-social history including relevant genetic information	Page #5, lines #86–95
	5d	Relevant past interventions with outcomes	Page #5, lines #86–95
**Clinical Findings**	6	Describe significant physical examination (PE) and important clinical findings.	Page #6, lines #96–107
**Timeline**	7	Historical and current information from this episode of care organized as a timeline	Page #6, lines #108–114
**Diagnostic Assessment**	8a	Diagnostic testing (such as PE, laboratory testing, imaging, surveys).	Page #6, lines #115–120
	8b	Diagnostic challenges (such as access to testing, financial, or cultural)	Page #6, lines #115–120
	8c	Diagnosis (including other diagnoses considered)	Page #6, lines #115–120
	8d	Prognosis (such as staging in oncology) where applicable	NA
**Therapeutic Intervention**	9a	Types of therapeutic intervention (such as pharmacologic, surgical, preventive, self-care)	Page #6–7, lines #115–122
	9b	Administration of therapeutic intervention (such as dosage, strength, duration)	Page #6–7, lines #115–122
	9c	Changes in therapeutic intervention (with rationale)	Page #6–7, lines #115–122
**Follow-up and Outcomes**	10a	Clinician and patient-assessed outcomes (if available)	Page #7, lines #122–123
	10b	Important follow-up diagnostic and other test results	Page #7, lines #123–126
	10c	Intervention adherence and tolerability (How was this assessed?)	Page #7, lines #122–123
	10d	Adverse and unanticipated events	Page #7, lines #122–123
**Discussion**	11a	A scientific discussion of the strengths AND limitations associated with this case report	Page #7–10, lines #128–201
	11b	Discussion of the relevant medical literature **with references**.	Page #7–9, lines #128–186
	11c	The scientific rationale for any conclusions (including assessment of possible causes)	Page #7–9, lines #128–194
	11d	The primary "take-away" lessons of this case report (without references) in a one paragraph conclusion	Page #10, lines #195–201
**Patient Perspective**	12	The patient should share their perspective in one to two paragraphs on the treatment(s) they received	NA
**Informed Consent**	13	Did the patient give informed consent? Please provide if requested	Yes ☑ No ☐
